# IgG4-related disease: a case report

**DOI:** 10.1515/almed-2019-0045

**Published:** 2020-03-20

**Authors:** Silvia de las Heras Flórez, Mercedes Carretero Pérez, Carmen Teresa Sanz Díaz, José Alejandro Medina García

**Affiliations:** Unidad Core, Hospital Universitario Nuestra Señora de Candelaria, Santa Cruz of Tenerife, Spain; Internal Medicine, Hospital Universitario Nuestra Señora de Candelaria, Santa Cruz of Tenerife, Spain; Nuestra Señora de la Candelaria University Hospital Ringgold standard institution - Core-Protein Unit, Carretera General del Rosario 145, 38010 Santa Cruz of Tenerife, Spain

**Keywords:** histopathological, IgG4, IgG4-related disease

## Abstract

IgG4-related disease (IgG4-RD) is a condition that was first described recently, and is capable of affecting any organ of the body. Diagnosis is based on the correlation of clinical findings with histopathological findings and elevated serum IgG4. Treatment involves corticosteroids and rituximab for the most severe cases. We report the case of a symptomatic patient diagnosed of IgG4-RD whose diagnosed was guided by elevated serum IgG4 levels.

## Introduction

IgG4-related disease (IgG4-RD) is a recently-described clinico-pathological condition with a wide range of clinical manifestations. Clinical signs include neoplasms, a dense lymphoplasmacytic infiltrate rich in IgG4-positive plasma cells, fibrosis arranged in a storiform pattern, obliterative phlebitis and, most commonly, elevated serum IgG4 concentrations [1]. The prevalence and incidence of the disease are poorly known, and they are probably underestimated.

This entity was first described in 2001, when autoimmune pancreatitis in a patient correlated with elevated serum IgG4 levels and numerous IgG4-rich lymphoplasmacytic cells [2]. In 2003, IgG4-RD was defined as a new systemic clinicopathological entity [3]. The term “IgG4-related disease” was coined in 2012 [4]. A large number of different diseases are now considered to be manifestations of IgG4-RD.

IgG4-RD is generally diagnosed when patients are in their 60s or 70s and is more frequent in men. The organ most commonly involves is the pancreas, although IgG4-RD can involve any site in the body [5], [6], [7], [8], including the salivary glands, kidneys, aorta, lungs, and retroperitoneum.

Presentation is subacute and patients rarely exhibit constitutional symptoms, with a predominance of symptoms of organ dysfunction. Patients commonly have a previous history of rhinitis, asthma, nasal polyps and athopy [9].

Diagnosis may be challenging as symptoms are nonspecific and are related with other more common diseases. Symptoms and laboratory findings may guide the clinician to further appropriate imaging studies and reach a diagnosis.

## Case presentation

A 29 year-old patient with a history of allergic rhinitis seen in Primary Care in December 2015 for a 6-month history of asthenia and a weight loss of 10 kg.

Physical examination was unremarkable. Laboratory tests showed biochemical alterations in blood, namely: elevated creatinine levels (1.95 mg/dL; reference range: 0.70–1.20 mg/dL), eosinophilia (3.51 × 103 cells/μL; reference cells: <0.50 cells/μL), elevated cholestatic enzymes gamma-glutamyl transferase (GGT) enzyme concentrations (263 U/L; reference range: 11–50 U/L) and alkaline phosphatase (523 UI/L; reference range: 40–129 UI/L), and a protein concentration of 9.2 g/dL (reference range: 6.6–8.7 g/dL). Although bilirubin was not tested, the index of jaundice was within normal limits.

Laboratory values and clinical symptoms led to patient's referral to the Outpatient Unit of Internal Medicine. Differential diagnosis with vasculitis, a lymphoproliferative and parasitic process was performed.

Laboratory findings included elevated IgG concentrations (4.132 mg/dL; reference range: 700–1.600 mg/dL) and IgE (1.281 UI/mL; reference range: <100 UI/mL). Other parameters were within normal limits, including autoimmunity markers (antinuclear antibodies, extractable nuclear antigen antibodies and neutrophil antithoplasma antibodies), serology (amoeba, Strongyloide, Trypanosoma Cruzi), CA19-9 (3.6 IU/mL; reference range: <37 UI/mL), adenosine deaminase, cryoglobulins, complement and Mantoux test.

The radiological study included a thoraco-abdomino-pelvic CT scan, which demonstrated multiple large adenopathies and a renal and periportal lymphomatous infiltration, with a lymphoproliferative process as first diagnostic option

Interconsultation with the Units of Nephrology and General Surgery was performed for a renal biopsy and surgical exeresis of an inguinal node for histopathological examination. Results were negative for a lymphoproliferative process, although they demonstrated significantly elevated lymphoplasmacytic cell levels. Samples were sent to the referring center for immunohistochemistry testing. The results obtained supported the suspicion of an IgG4-related disease.

Testing for IgG subclass levels yielded a total IgG concentration of 2.225 mg/dL, IgG1 of 1.432 mg/dL (reference range: 382–929 mg/dL); IgG2 of 816 mg/dL (reference range: 242–700 mg/dL); IgG3 of 184 mg/dL (reference range: 22–176 mg/dL) e IgG4: 652 mg/dL (reference range: 4–86 mg/dL).

Final diagnosis was performed based on the finding of very elevated IgG4 levels and after other pathologies had been excluded.

During the study period, three bolus units of methylprednisolone 125 mg and oral prednisone (10 mg/day) were administered. When diagnosis of IgG4-RD was confirmed, rituximab therapy was started in November 2016 (2 cycles of 1 g at 6-month intervals). The reason for this therapy was that clinical response to corticosteroids was only partial and resulted in the activation of herpes zoster, high blood pressure and cushingoid habitus. In May 2017, the other two cycles were administered.

The clinical status of the patient improved progressively, creatinine returned to normal levels and IgG4 was reduced to values below 135 mg/dL after the first four cycles of rituximab. Adenopathies and infiltration decreased in size.

## Discussion

IgG4-RD is a recently described entity with a challenging diagnosis. This disease is increasingly considered in the differential diagnosis of a variety of symptoms of organ or systemic dysfunction [10], [11].

The etiopathogenesis of the disease and the role of IgG4 are unclear, being the hypothesis of an autoimmune disease the most widely accepted [12].

Final diagnosis is based on the immunohistochemical analysis of a biopsy of the organ involved. However, laboratory plays a crucial role in the diagnosis of IgG4-RD. Thus, a range of non-validated diagnostic criteria have been proposed by different research groups, where IgG4 levels >135 mg/dL are highly suggestive of IgG4-RD [6], [12], [13].

Elevated serum IgG4 levels are considered to have high sensitivity and positive predictive value (>90%), but with a low specificity and negative predictive value [14]. Diagnostic determination of serum IgG4 levels for the diagnosis of this disease has some limitations, as IgG4 levels are not always elevated, and elevation of IgG4 is also associated with other entities such as bronchiectasis, asthma, sarcoidosis and adenocarcinoma of the pancreas, among others.

Serum IgG4 levels are also useful in monitoring patient's response to therapy, although inconsistent results have been published on this use of IgG4 levels [15], [16]. In this case, IgG4 levels were consistent with clinical evolution and response to therapy, although conclusive scientific evidence has not been provided to support this use.

The etiopathogenesis and the role of IgG4 in this disease are not clearly understood. The diagnostic value of serum IgG4 levels has not been sufficiently proven. However, its use in the adequate clinical setting, especially if concentrations double the reference limit can be useful to guide the clinician to further appropriate imaging studies and reach a diagnosis. Further evidence is needed to support the recommendation of using serum IgG4 levels as a prognostic marker and as an indicator of response to therapy.

Although the diagnostic value of other biomarkers is under study, further studies are needed to provide robust evidence [17].

The patient was young and exhibited nonspecific clinical symptoms, and histopathological and laboratory findings were crucial to obtaining the diagnosis of IgG4-RD after only 6 months after the onset of clinical symptoms. The mean time to diagnosis is 4–5 years, especially as this entity mimics other diseases. Fortunately, IgG4-RD is increasingly included in differential diagnosis.
